# Pharmacological inhibition of SMYD2 protects against cisplatin-induced acute kidney injury in mice

**DOI:** 10.3389/fphar.2022.829630

**Published:** 2022-08-15

**Authors:** Binbin Cui, Xiying Hou, Mengjun Liu, Qing Li, Chao Yu, Shenglei Zhang, Yi Wang, Jun Wang, Shougang Zhuang, Feng Liu

**Affiliations:** ^1^ Department of Nephrology, Shanghai East Hospital, Tongji University School of Medicine, Shanghai, China; ^2^ Department of Pathology, Shanghai East Hospital, Tongji University School of Medicine, Shanghai, China; ^3^ Department of Medicine, Rhode Island Hospital and Alpert Medical School, Brown University, Providence, RI, United States

**Keywords:** acute kidney injury, SMYD2, kidney tubular cells, apoptosis, inflammation, proliferation, p53, cisplatin

## Abstract

The histone methyltransferase SET and MYND domain protein 2 (SMYD2) has been implicated in tumorigenesis through methylating histone H3 at lysine36 (H3K36) and some non-histone substrates. Currently, the role of SMYD2 in acute kidney injury (AKI) remains unknown. Here, we investigated the effects of AZ505, a highly selective inhibitor of SMYD2, on the development of AKI and the mechanisms involved in a murine model of cisplatin-induced AKI. SMYD2 and trimethylated histone H3K36 (H3K36Me3) were highly expressed in the kidney following cisplatin treatment; administration of AZ505 remarkedly inhibited their expression, along with improving kidney function and ameliorating kidney damage. AZ505 also attenuated kidney tubular cell injury and apoptosis as evidenced by diminished the expression of neutrophil gelatinase associated lipocalin (NGAL) and kidney injury molecule (Kim-1), reduced the number of TUNEL positive cells, decreased the expression of cleaved caspase-3 and the BAX/BCL-2 ratio in injured kidneys. Moreover, AZ505 inhibited cisplatin-induced phosphorylation of p53, a key driver of kidney cell apoptosis and reduced expression of p21, a cell cycle inhibitor. Meanwhile, AZ505 promoted expression of proliferating cell nuclear antigen and cyclin D1, two markers of cell proliferation. Furthermore, AZ505 was effective in suppressing the phosphorylation of STAT3 and NF-κB, two transcriptional factors associated with kidney inflammation, attenuating the expression of monocyte chemoattractant protein-1 and intercellular cell adhesion molecule-1 and reducing infiltration of F4/80^+^ macrophages to the injured kidney. Finally, in cultured HK-2 cells, silencing of SMYD2 by specific siRNA inhibited cisplatin-induced apoptosis of kidney tubular epithelial cells. Collectively, these results suggests that SMYD2 is a key determinant of cisplatin nephrotoxicity and targeting SMYD2 protects against cisplatin-induced AKI by inhibiting apoptosis and inflammation and promoting cell proliferation.

## Introduction

Acute kidney injury (AKI) can be induced by a variety of insults, including ischemia/reperfusion, sepsis, rhabdomyolysis and nephrotoxicants (Peerapornratana et al., 2019; Ronco et al., 2019). Nephrotoxic agent-induced AKI affects approximately one-third of patients with AKI in clinical practice (Xiang et al., 2019). Among nephrotoxic agents that cause AKI, cisplatin is a chemotherapeutic drug that has been extensively used for treating solid cancers including breast, pancreatic, colorectal, esophageal, blood, lung, bladder, and hepatocellular cancers (Holditch et al., 2019; Rottenberg et al., 2021). Kidney uptake and excretion of cisplatin mainly occurs in proximal tubules (Perazella, 2019). Multiple and long-term use of cisplatin and other platinum compounds causes accumulation in kidney tubular cells, resulting in cell injury and apoptosis as well as kidney inflammation (Manohar and Leung, 2018; Perše and Večerić-Haler, 2018). So far, the molecular mechanisms of cisplatin-induced AKI remain incompletely understood, and there are no effective therapeutic approaches to protect against its nephrotoxicity.

Accumulating data indicate that epigenetic modifications such as histone methylation contributes to AKI induced by multiple insults, including nephrotoxicants (Zhuang, 2018; Xiang et al., 2019). Histone methylation can change gene transcription through creating docking sites for chromatin modifiers (Guo et al., 2019) and is subject to regulation by histone methyltransferases and demethylases (Fontecha-Ba rriuso et al., 2018). Many histone methyltransferases and demethylases have been identified and implicated in the diverse pathological processes, including kidney diseases (Li et al., 2019). Among histone methyltransferases, ours and other studies have identified SET and MYND domain protein 2 (SMYD2) as a critical driver in cystic growth and kidney fibrosis in murine models (Li et al., 2017; Liu et al., 2021). However, the role of SMYD2 in AKI remains unexplored.

SMYD2 belongs to the SMYD family that is composed of five members (SMYD1-5) (Weirich et al., 2020). Among them, SMYD1 and SMYD2 are the two most studied isoforms (Rueda-Robles et al., 2021). While SMYD1 is uniquely expressed in the heart and muscle, SMYD2 is expressed in multiple mammalian tissues including the kidney during development (Jarrell et al., 2020). SMYD2 exerts its biological functions mainly through methylation of histone H3K36 and some non-histone proteins such as p53, phosphatase and tensin homolog deleted on chromosome 10 (PTEN), signal transducer and activator of transcription 3 (STAT3), and nuclear factor kappa-light-chain-enhancer of activated B cells (NF-κB) (Yi et al., 2019; Weirich et al., 2020; Rueda-Robles et al., 2021). All of these non-histone proteins are involved in processes associated with the pathogenesis of AKI (Holditch et al., 2019; Uber and Sutherland, 2020). Since SMYD2 is over-expressed in multiple types of solid tumors, and cisplatin remains among the most widely used anticancer drugs. A recent study examined the role of SMYD2 in chemotherapeutic resistance (Shang and Wei, 2019). The results demonstrated that inhibition of SMYD2 by a specific inhibitor enhanced cell sensitivity to cisplatin in non-small cell lung cancer (NSCLC) (Shang and Wei, 2019), suggesting that the combination of SMYD2 inhibitor and cisplatin may be a novel treatment for patients with cisplatin-resistant NSCLC (Shang and Wei, 2019). On this basis, it will be interesting to examine whether inhibition of SMYD2 would also affect the cisplatin nephrotoxicity.

In this study, we investigated the effect of SMYD2 inhibition on the development of AKI induced by cisplatin in a mouse model by using AZ505, a substrate-competitive inhibitor that binds the peptide-bind in groove of SMYD2 to reduce its methyltransferase activity (Ferguson et al., 2011). We found that treatment with AZ505 is protective against cisplatin-induced AKI through mechanisms involved in inhibiting apoptosis and inflammation, and promoting kidney tubular cell proliferation. In addition, we found that siRNA-mediated silencing of SMYD2 reduced cisplatin-induced injury of cultured kidney tubular cells.

## Results

### Administration of AZ505 inhibits expression of SMYD2 and H3K36me3 in the kidney of mice following cisplatin treatment

**FIGURE 1:**
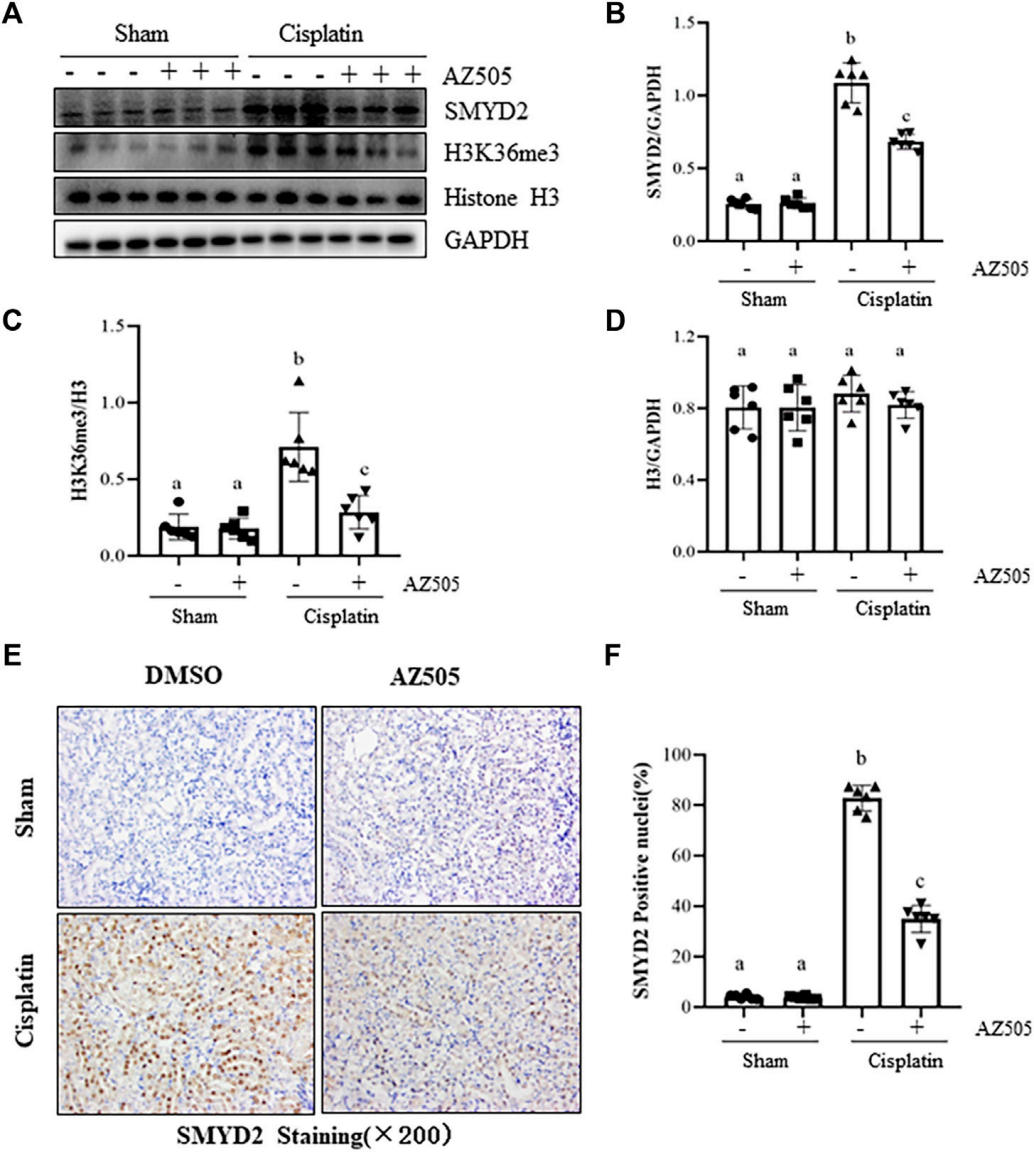
Administration of AZ505 inhibits cisplatin-induced expression and activation of SMYD2. Mice received cisplatin-injection and AZ505 administration as described in Materials and Methods. **(A)** The kidney tissues were taken for immunoblot analysis of SMYD2, H3K36me3, Histone H3 and GAPDH as indicated. Representative immunoblots from 3 experiments are shown. **(B–D)** Expression levels of SMYD2 **(B)**, and Histone H3 **(D)** were quantified by densitometry and normalized with GAPDH,along with H3K36me3 **(C)** normalized with Histone H3 as indicated. **(E)** Photomicrographs illustrate immunohistochemical staining for SMYD2 in the kidney tissues with or without AZ505 treatment (×200). **(F) **The graph shows the percentage of SMYD2-positive nucleis (brown) relative to the whole nucleis from 10 random cortical fields (×200). Data are means ± S.E.M. (*n* = 6). Means with different superscript letters are significantly different from one another (*p* < 0.05).

To determine the role of SMYD2 in AKI, we first examined the expression of SMYD2 and its epigenetic marker H3K36me3, and the impact of AZ505, a highly selective inhibitor of SMYD2 in a mouse model of cisplatin-induced AKI. Immunoblot analysis indicated that SMYD2 and H3K36me3 were minimally expressed in the sham kidney, but their levels were markedly increased in the kidney following cisplatin treatment. Administration of AZ505 at 10 mg/kg did not affect basal levels of SMYD2 and H3K36me3, but largely reduced cisplatin-induced upregulation of SMYD2 and H3K36me3 without altering expression of histone H3 (Figures 1A–D). Immunohistochemical staining demonstrated that SMYD2 was barely detected in the sham operated kidney, and its expression increased significantly after cisplatin injection; AZ505 was effective in suppressing SMYD2 expression (Figures 1E,F). Notably, SMYD2 was located in both the cytosol and nucleus of kidney tubular epithelial cells (TECs). These data indicated that cisplatin at toxic dose enhances SMYD2 expression and activation in the kidney, which was sensitive to AZ505 treatment.

### Inhibition of SMYD2 with AZ505 improves kidney function and ameliorates kidney pathological damage induced by cisplatin

**FIGURE 2:**
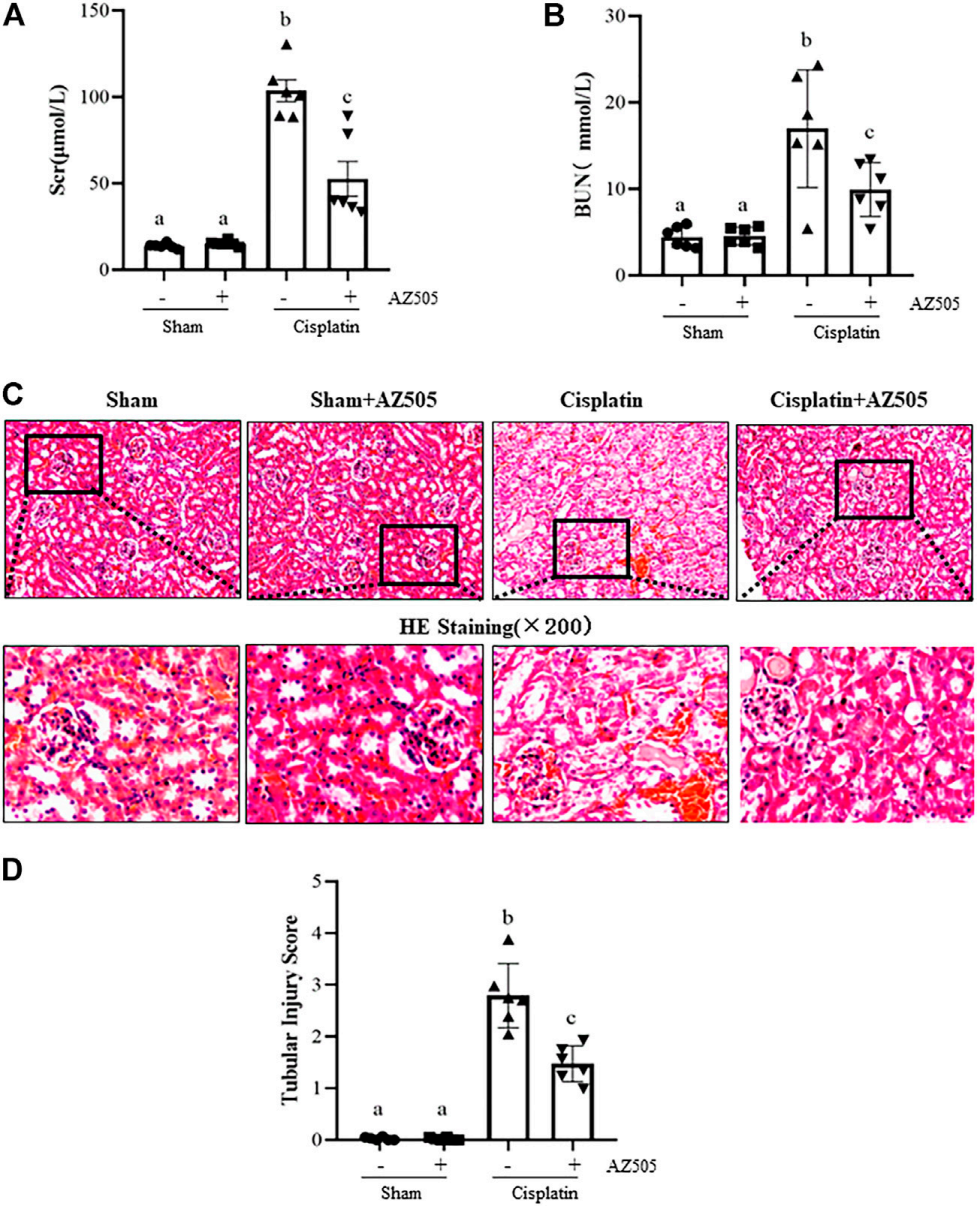
Inhibition of SMYD2 with AZ505 improves kidney function and ameliorates kidney pathological damage induced by cisplatin. **(A,B)** Mice blood was collected at 48h after cisplatin injection with or without AZ505 administration, Scr **(A)** and BUN **(B)** were measured by assays as indicated in the Materials and Methods. **(C)** Photomicrographs illustrate HE staining of the kidney tissues with or without AZ505 treatment (×200). **(D)** The graph shows the scores of kidney tubular injury in each group based on the scale described in Materials and Methods. Data are means ± S.E.M. (*n* = 6). Means with different superscript letters are significantly different from one another (*p* < 0.05).

At 48 h following cisplatin injection, blood was collected, and serum creatinine (Scr) and blood urea nitrogen (BUN) were measured and renal histopathology was examined. Figure 2 showed that both Scr and BUN were elevated and pathological changes (i.e., tubular distention, swelling and necrosis and preservation of a brush border) were observed in the kidney after cisplatin injection. Administration of AZ505 significantly reduced Scr and BUN levels (Figures 2A,B) and alleviated cisplatin-induced kidney tubular damage (Figures 2C,D). AZ505 alone neither affected kidney function nor caused pathological damage to the kidney. These results suggested that targeting SMYD2 by AZ505 can protect against cisplatin-induced AKI.

### Administration of AZ505 attenuates cisplatin-induced kidney tubular injury

**FIGURE 3:**
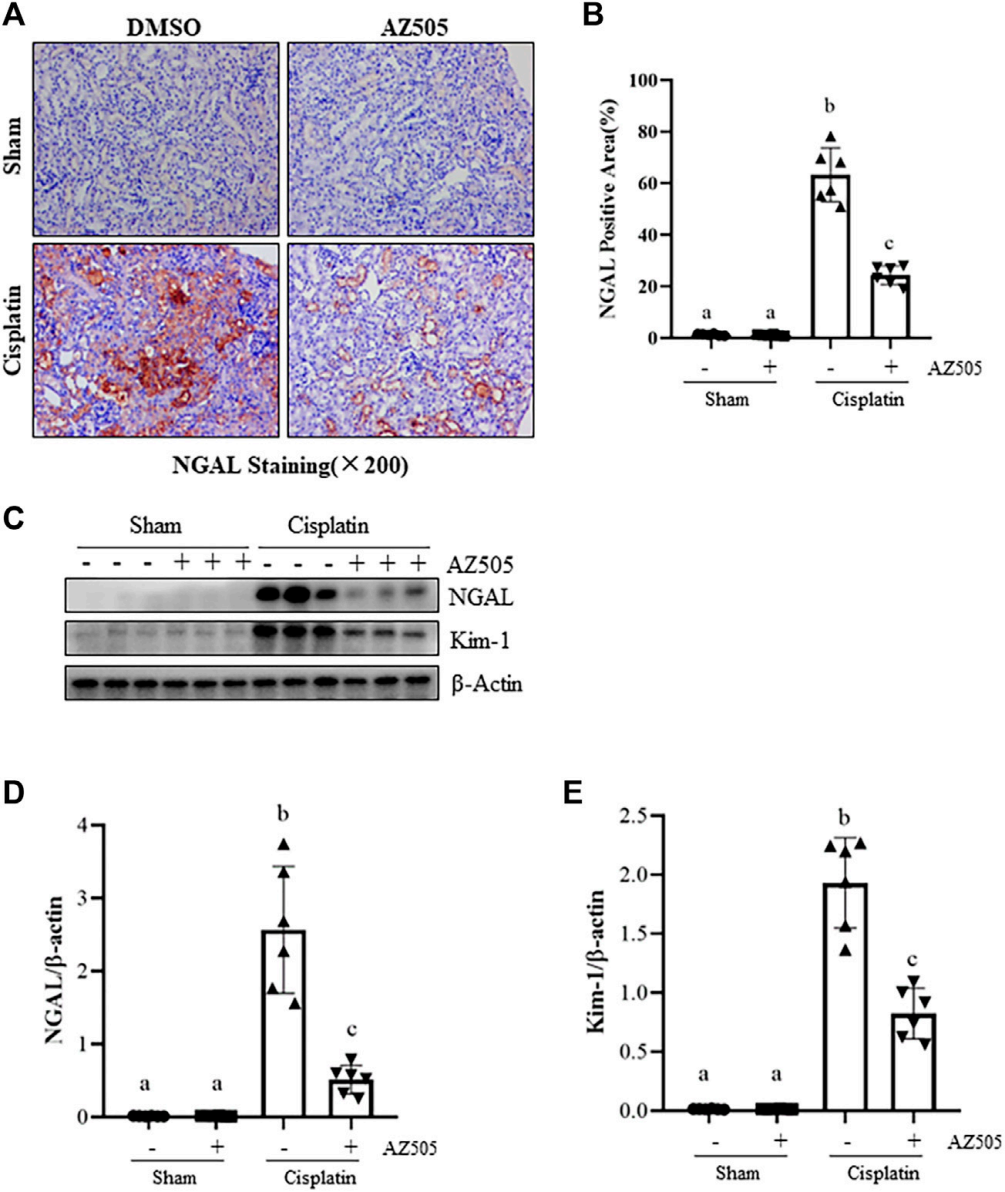
Administration of AZ505 ameliorates cisplatin-induced kidney tubular injury. **(A)** Photomicrographs illustrate immunohistochemical staining for NGAL of the kidney tissues with or without AZ505 treatment (×200). **(B)** The graph shows the percentage of NGAL-positive areas (brown) relative to the whole area from 10 random cortical fields (×200). **(C)** The kidney tissues were taken for immunoblot analysis of NGAL, Kim-1 and β-Actin as indicated. Representative immunoblots from 3 experiments are shown. **(D,E)** Expression levels of NGAL **(D)** and Kim-1 **(E)** were quantified by densitometry and normalized with β-Actin as indicated. Data are means ± S.E.M (*n* = 6). Means with different superscript letters are significantly different from one another (*p* < 0.05).

To further determine the role of SMYD2 in cisplatin-induced kidney tubular injury, we examined the expression of kidney injury biomarkers, neutrophil gelatinase-associated lipocalin (NGAL) and kidney injury molecule (Kim-1) by immunohistochemistry and immunoblot analysis. As shown in Figures 3A,B, NGAL was not expressed in the sham kidney with/without AZ505 administration, but its expression levels were markedly increased in the kidney following cisplatin treatment; AZ505 treatment largely reduced its expression. Immunoblot analysis confirmed the inhibitory effect of AZ505 on the expression of NGAL and Kim-1 in the cisplatin-treated kidney (Figures 3C,D). Therefore, these results illustrated that inhibition of SMYD2 by AZ505 could effectively alleviate kidney tubular injury induced by cisplatin in mice.

### Administration of AZ505 inhibits cisplatin-induced kidney tubular cell apoptosis in the kidney following cisplatin treatment

**FIGURE 4:**
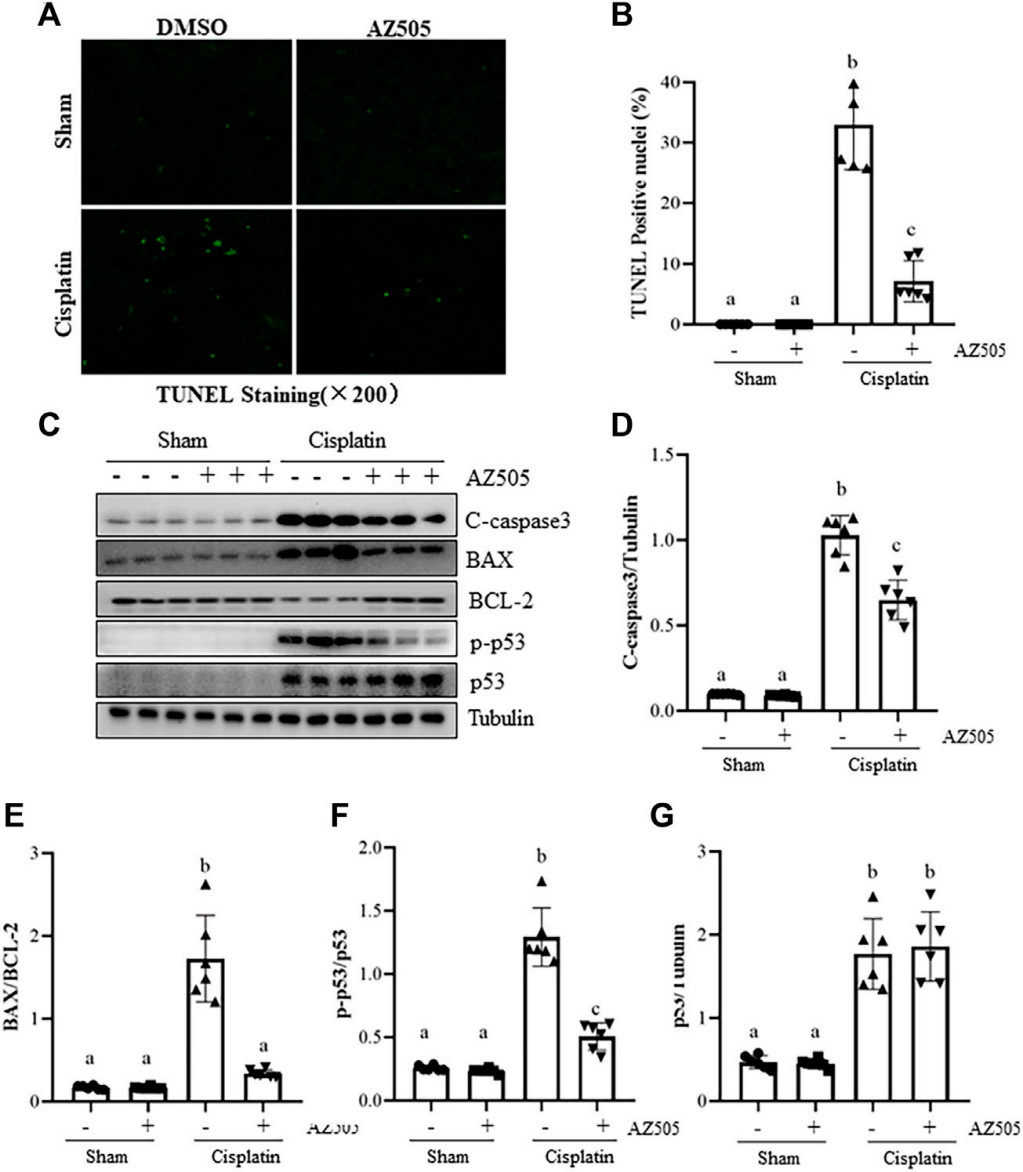
Administration of AZ505 reduces kidney tubular cells apoptosis induced by cisplatin. Photomicrographs illustrate TUNEL staining of the kidney tissues collected at 48 h after cisplatin injection with or without AZ505 administration. **(B)** The number of positive TUNEL staining cells were counted in 10 high-power fields (×200). **(C)** The kidney tissues were taken for immunoblot analysis of cleaved-Caspase-3, BAX, BCL-2, phospho-p53 (p-p53), total p53 and Tubulin as indicated. Representative immunoblots from 3 experiments are shown. **(D–G)** Expression levels of cleaved-Caspase-3**(D)**, BAX/BCL-2 ratio **(E)** and p53 **(G)** were quantified by densitometry and normalized with β-Actin, along with p-p53 **(F)** normalized with total p53 as indicated. Data are means ± S.E.M. (*n* = 6). Means with different superscript letters are significantly different from one another (*p* < 0.05).

Kidney tubular cell apoptosis is a predominant feature in cisplatin-induced AKI (Holditch et al., 2019). To investigate whether SMYD2 activation contributes to this process, we first examined the effect of AZ505 on apoptosis, cleavage of caspase-3, expression of B-cell lymphoma-2 (BCL-2) and BCL-2 associated X protein (BAX). Immunostaining displayed a large number of TUNEL-positive cells in cisplatin-injured kidneys whereas AZ505 administration significantly diminished this population of cells (Figures 4A,B). Immunoblot analysis demonstrated that cisplatin induced cleavage of caspase-3, upregulation of BAX and downregulation of BCL-2 in the kidney; AZ505 treatment also significantly reversed these responses, in particular, the BAX/BCL-2 ratio. Notably, there was a basal level of cleaved caspase-3, BAX and BCL-2 in sham-operated kidneys.

As a potential non-histone substrate of SMYD2, activated p53 interacts with specific DNA sequences that promote the transcription of certain genes related to apoptosis caused by cisplatin (Holditch et al., 2019). We found that phospho-p53 was minimally expressed in the sham kidney, and expression of phospho-p53 and total p53 were increased after cisplatin injection. AZ505 administration significantly reduced p53 phosphorylation, but did not alter the expression of total p53 protein (Figures 4C–G).

Collectively, these results implicated that AZ505 treatment may be protective against cisplatin-induced AKI by suppression of caspase-3 cleavage, reducing the BAX/BCL-2 ratio and inhibition of p53 phosphorylation.

### Administration of AZ505 promotes kidney tubular cell proliferation and cell cycle progression in the kidney following cisplatin treatment

**FIGURE 5:**
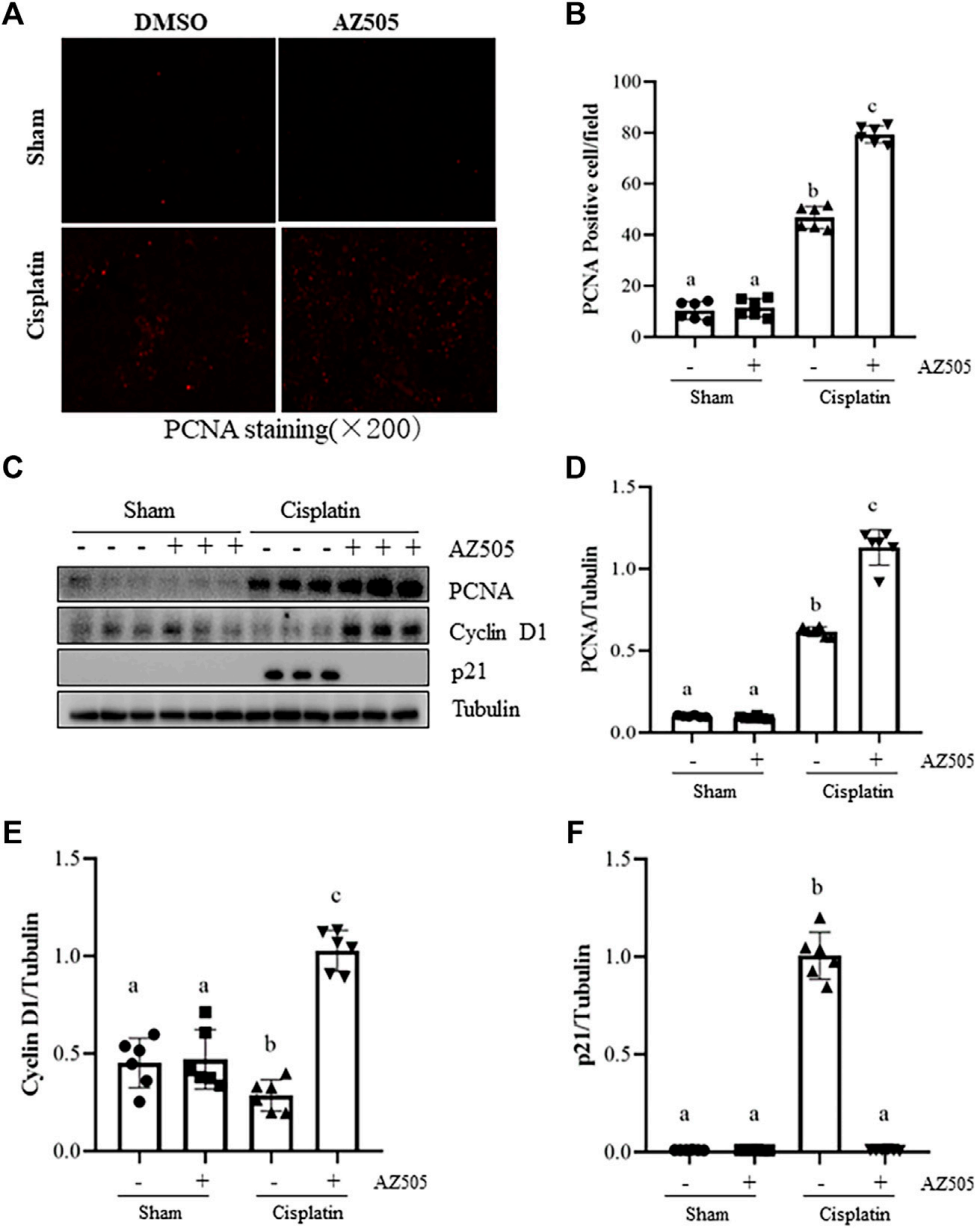
Administration of AZ505 promotes kidney tubular cell proliferation during the progression of cisplatin-induced AKI. **(A)** Photomicrographs illustrate immunofluorescence staining for PCNA of the kidney tissues with or without AZ505 treatment (×200). **(B)** The graph shows the percentage of PCNA-positive areas (red) relative to the whole area from 10 random cortical fields (×200). **(C)** The kidney tissues were taken for immunoblot analysis of PCNA, cyclin D1, p21 and Tubulin as indicated. Representative immunoblots from 3 experiments are shown. **(D–F)** Expression levels of PCNA **(D)**, cyclin D1 **(E)** and p21 **(F)** were quantified by densitometry and normalized with β-Actin as indicated. Data are means ± S.E.M. (*n* = 6). Means with different superscript letters are significantly different from one another (*p* < 0.05).

Proliferation of kidney tubular cells is essential for kidney regeneration following kidney injury (Ronco et al., 2019). To determine the role of SMYD2 in this process, we examined the effect of AZ505 on the proliferation of kidney tubular cells. Our results from immunofluorescence staining showed that kidney tubular cells with positive proliferating cell nuclear antigen (PCNA, a marker of cell proliferation) was rarely observed in sham-operated kidneys with/without AZ505 administration. This population of cells was slightly increased in the kidney of AKI following cisplatin treatment while AZ505 treatment promoted this response (Figures 5A,B). In agreement with these results, immunoblot analysis demonstrated an increase in PCNA protein levels in the kidney following cisplatin treatment relative to that in control kidneys and AZ505 treatment enhanced PCNA expression in the injured kidney (Figures 5C,D).

Progression of cell cycle in TECs is regulated by cyclins, cyclin dependent kinases and cyclin-dependent kinase inhibitors. Cyclin D1 is a key protein that drives cell cycle progression while p21 is a potent cyclin-dependent kinase inhibitor that inhibits cell cycle progression (Karimian et al., 2016). Since SMYD2 has emerged as an important regulator of cell cycle, we next examined the effect of AZ505 on expression of these proteins. Injury to the kidney induced by cisplatin reduced the expression of cyclin D1 whereas AZ505 treatment restored its expression. In contrast, p21 was increased in the cisplatin-injured kidneys, and AZ505 was effective in reducing its expression (Figures 5C,E,F).

Taken together, AZ505 administration was able to promote a regenerative response of TECs to the injury initiated by cisplatin treatment.

### Administration of AZ505 suppresses cisplatin-induced expression of inflammatory cytokines and infiltration of inflammatory cells into injured kidneys

**FIGURE 6:**
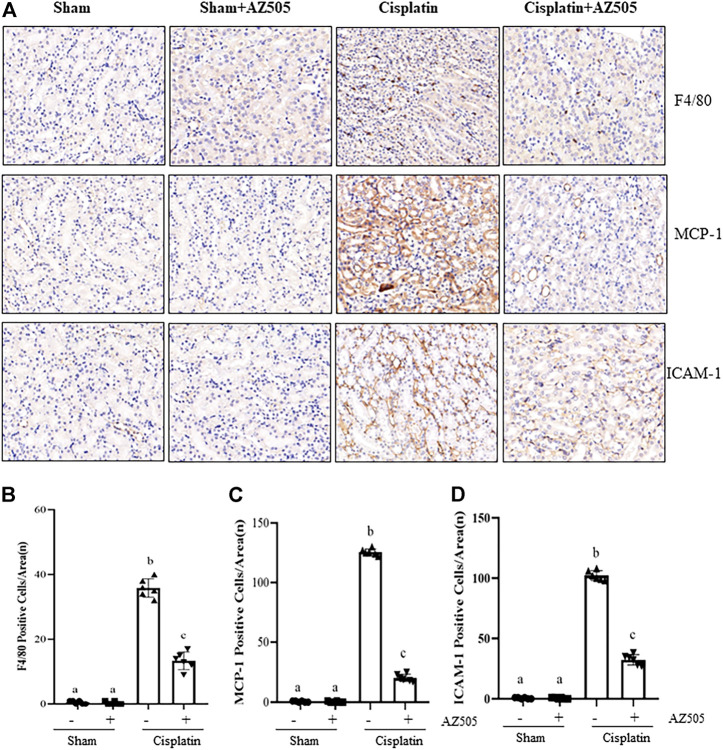
Administration of AZ505 suppresses cisplatin-induced inflammation in the kidney following cisplatin treatment. **(A)** Photomicrographs illustrate immunohistochemistry staining of F4/80-positive, MCP-1-positve and ICAM-1-positive cells in the kidney tissues treated with or without AZ505. **(B–D)** The graph shows the number of F4/80-positive macrophages **(B)**, MCP-1-positve cells **(C)** and ICAM-1-positive cells **(D)** were calculated from 10 random fields (200×). Data are means ± S.E.M. (*n* = 6). Means with different superscript letters are significantly different from one another (*p* < 0.05).

Renal inflammation is a common pathological change in AKI (Holditch et al., 2019). Our immunohistochemical staining showed that the number of F4/80-positive macrophages were increased in the cisplatin-injured kidney compared with sham kidneys, and administration of AZ505 significantly reduced macrophage infiltration (Figures 6A,B). AZ505 also largely attenuated the elevated expression of monocyte chemoattractant protein-1 (MCP-1) and intercellular cell adhesion molecule-1 (ICAM-1) in the kidney following cisplatin treatment (Figures 6A,C,D). Thus, our results suggested that inhibition of SMYD2 by AZ505 may suppress the inflammatory response in cisplatin-induced AKI.

### Administration of AZ505 blocks cisplatin-induced phosphorylation of STAT3 and NF-κB and upregulates PTEN activation

**FIGURE 7:**
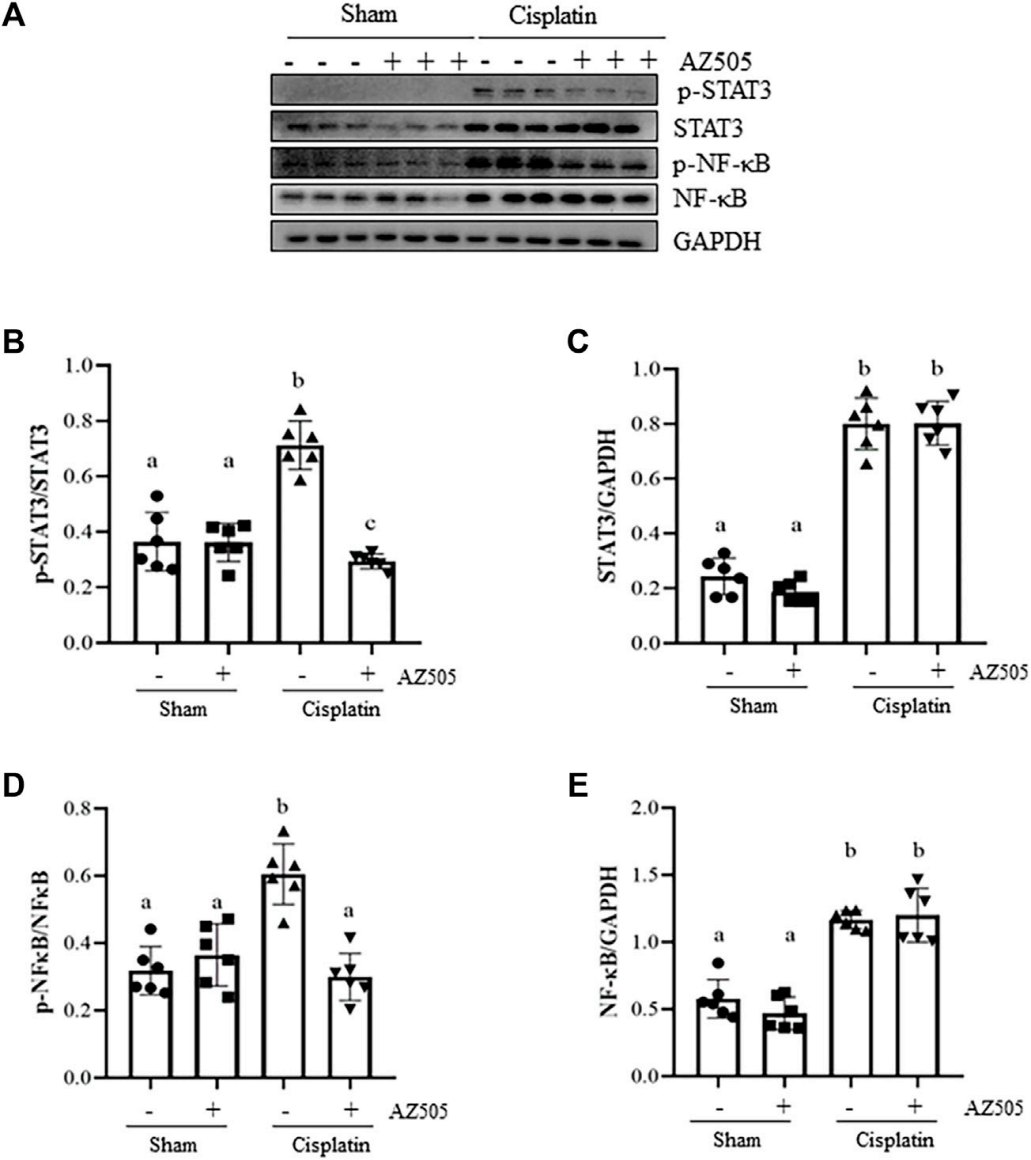
Administration of AZ505 blocks phosphorylation of STAT3 and NF-κB induced by cisplatin. **(A)** The kidney tissues were taken for immunoblot analysis of phospho-STAT3 (p-STAT3), phospho-NF-κB (p-NF-κB), total STAT3, total NF-κB, and GAPDH as indicated. Representative immunoblots from 3 experiments are shown. **(B,D)** Expression levels of p-STAT3 **(B)** and p-NF-κB **(D)** were quantified by densitometry and normalized with total STAT3 and NF-κB as indicated, respectively. **(C,E)** Expression levels of total STAT3 **(C)** and NF-κB **(E)** was quantified by densitometry and normalized with GAPDH as indicated, respectively. Data are means ± S.E.M. (n = 6). Means with different superscript letters are significantly different from one another (*p* < 0.05).

Since STAT3 and NF-κB signaling pathways are critically involved in production of multiple inflammatory cytokine and chemokines in AKI, we further explored whether SMYD2 activity would be required for the activation of these two signaling pathways in the kidney following cisplatin exposure. As shown in Figures 7A–F, significant increasement of STAT3 and NF-κB phosphorylation was observed in the kidney exposed to cisplatin. Administration of AZ505 reduced cisplatin-induced phosphorylation of STAT3 and NF-κB, whereas the expression levels of total STAT3 and NF-κB remained the same in all the groups (Figures 7A,C,E). Thus, our results demonstrated that blocking SMYD2 by AZ505 inhibits cisplatin-induced activation of these two cellular signaling pathways.

### Silencing of SMYD2 by specific siRNA inhibits cisplatin-induced injury and apoptosis of kidney tubular cells *in vitro*.

**FIGURE 8:**
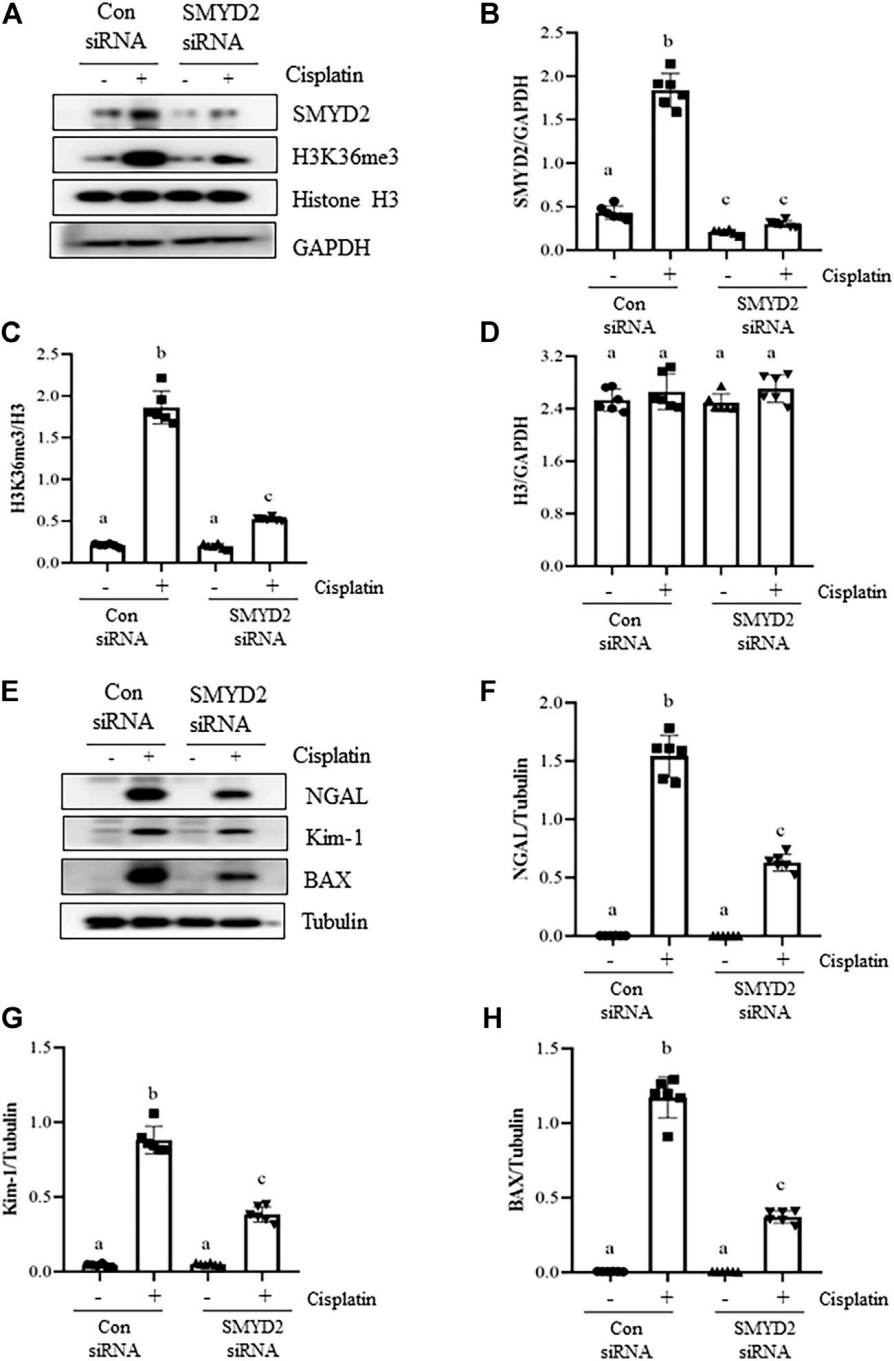
Silencing of SMYD2 by specific siRNA inhibits cisplatin-induced injury and apoptosis of kidney tubular cells *in vitro*. **(A)** Cell lysates were prepared for immunoblot analysis with antibodies against SMYD2, H3K36me3, Histone H3 and GAPDH. **(B–D)** Expression levels of SMYD2 **(B)**, and Histone H3 **(D)** were quantified by densitometry and normalized with GAPDH,along with H3K36me3 **(C)** normalized with Histone H3 as indicated. **(E)** Cell lysates were prepared for immunoblot analysis with antibodies against NGAL, Kim-1, BAX and Tubulin. **(F–H)** Expression levels of NGAL **(F)**, Kim-1 **(G)** and BAX **(H)** were quantified by densitometry and normalized with Tubulin as indicated. Data are means ± S.E.M. (*n* = 6). Means with different superscript letters are significantly different from one another (*p* < 0.05).

To verify the regulatory role of SMYD2 in TECs, we also examined the effect of siRNA-mediated SMYD2 silencing on the expression of SMYD2, H3K36me3, the marker proteins of injury (NGAL, Kim-1) and apoptosis (BAX) in HK-2 cells following cisplatin exposure. As shown in Figures 8A–D, similar to the inhibitory effect of AZ505 on SMYD2, transfection of cells with SYMD2 siRNA largely suppressed SMYD2 and H3K36me3 expression in cisplatin-treated cells. Moreover, as shown in Figures 8E–H, immunoblot analysis demonstrated the inhibitory effect of SMYD2 siRNA on the expression of NGAL, Kim-1 and BAX in the cisplatin-treated HK-2 cells. These results further provide evidence that SMYD2 is involved in cisplatin-induced kidney tubular cell injury and apoptosis.

## Discussion

SMYD2 has been reported to be implicated in the development of cardiovascular disease, cancer, and autosomal dominant polycystic kidney disease (ADPKD) (Li et al., 2017; Tracy et al., 2018; Yi et al., 2019; Rueda-Robles et al., 2021). Recently, we also demonstrated that SMYD2 contributes to kidney fibrosis in a murine model of UUO (Liu et al., 2021). In this study, we found that kidney SMYD2 expression and its activity were increased in a murine model of cisplatin-induced AKI and that inhibition of SMYD2 by AZ505 improves renal function, attenuates kidney tubular injury and inflammation, and promotes kidney tubular cell proliferation. These data indicated that SMYD2 activation contributes to the pathogenesis of AKI induced by cisplatin and suggested that SMYD2 might be a potential therapeutic target for the treatment of AKI induced by cisplatin.

SMYD2 is a histone methyltransferase that contains a SET domain and a MYND domain (Rueda-Robles et al., 2021). The SET domain has lysine-specific methyltransferase activity, while the MYND domain contains a zinc-finger motif able to mediate protein-protein interactions (Yi et al., 2019). Thus, SMYD2 has an ability to methylate histones and non-histone proteins (Weirich et al., 2020). Indeed, studies have demonstrated that SMYD2 can catalyze methylation of H3K36 and some non-histone proteins such as p53, PTEN, cyclin D1, STAT3, p65NFκB, and mitogen-activated protein kinase-activated protein kinase 3 (MAPK-APK3) (Weirich et al., 2020). In agreement with these observations, we found that following cisplatin treatment, SMYD2 was highly expressed in the kidney and in the cultured kidney tubular cells, and distributed in both nucleus and cytosol of TECs; blocking SMYD2 with AZ505 not only inhibited expression of H3K36me3, but also phosphorylation of p53 at Ser 15, STAT3 at Ser 705 and NF-κB at Ser310, in kidneys exposed to cisplatin. Meanwhile, silencing of SMYD2 with siRNA also suppressed SMYD2 and H3K36me3 expression in cisplatin-treated kidney tubular cells. This suggests that SMYD2-mediated methylation of these proteins can affect their phosphorylation. Since p53, STAT3 and NF-κB phosphorylation at individual sites is associated with their activation and the pathogenesis of AKI, it is anticipated that administration with AZ505 may improve kidney function and minimize tubular cell injury through inhibiting H3K36me3-mediated gene transcription as well as regulating phosphorylation of these non-histone proteins (see below). In addition, since p53, STAT3 and NF-κB are transcriptional factors that can regulate expression of multiple genes (Tang et al., 2019; Zhu et al., 2020), it is also possible that SMYD2-induced activation of these and/or other signaling pathway might regulate expression of SMYD2 itself through a feed-back mechanism, while AZ505 treatment may block this process, leading to downregulation of their expression.

Our data suggests that SMYD2 is an important mediator of kidney tubular cell apoptosis during cisplatin-induced AKI. This is supported by our observations that either blocking SMYD2 with AZ505 or silencing SMYD2 with siRNA inhibited cisplatin-induced expression of NGAL and Kim-1 in kidney tubular cells, and AZ505 significantly reduced the number of TUNEL positive tubular cells. Moreover, AZ505 was effective in suppressing cleavage of caspase-3, a key enzyme responsible for the execution of apoptosis in the cisplatin-induced injured kidney. Given caspase-3 activation is secondary to cytochrome C release resultant from mitochondria damage (Tonnus et al., 2019). The imbalance of BAX and BCL-2 expression contributes to the permeabilization of mitochondrial membrane and release of cytochrome C, ultimately resulting in apoptosis through caspase activation (Holditch et al., 2019; Tonnus et al., 2019). Therefore, we also examined the effect of SMYD2 inhibition on the expression ratio of BAX/BCL-2. Interestingly, inhibition of SMYD2 by either AZ505 or SMYD2 siRNA inhibited cisplatin-induced BAX upregulation, and AZ505 also restored the downregulated BCL-2 in cisplatin exposed kidneys, suggesting that an increase in the BAX/BCL-2 ratio may serve as a mediator of SMYD2 activation to caspase-3 cleavage and apoptosis.

It remains elusive how SMYD2 contributes to BAX expression and/or activation. Previous studies have showed that BAX is subjected to the regulation by p53 while p53 activation is an important mediator of apoptosis in response to cisplatin treatment (Holditch et al., 2019). Moreover, BAX can be regulated by p53 in transcriptional-dependent and independent mechanisms (Miyashita and Reed, 1995; Chipuk et al., 2004). In this study, we observed that inhibition of SMYD2 by AZ505 inhibited p53 phosphorylation, coincident with its inhibition of BAX. This suggests that cisplatin-induced activation of SMYD2 may lead to p53 methylation and activation, and then triggers BAX activation and apoptosis. In this context, SMYD2 was reported to be able to induce its mono-methylation on lysine 370 residues (Chandramouli et al., 2019), however, SMYD2 mono-methylation is repressive to p53-mediated transcriptional regulation and apoptosis, and p53 phosphorylation at serine 15 was not affected by SMYD2 (Huang et al., 2006). These results are in contrast to our observations. The underlying mechanism for these differences is unclear but may involve other post-translational modifications on p53 or demethylase-associated proteins. For example, SMYD2 may not only regulate p53 activity by direct methylation, but also indirectly regulate its activity by inducing expression of other methyltransferases or demethylases that regulate p53 methylation (Chandramouli et al., 2019). In this regard, although SMYD2 is documented to be a monomethyl transferase, both K370me1 and K370me2 of p53 are detected in cells. Moreover, K370me2, in contrast to K370me1, has an activating role in p53 regulation through promoting the interaction of p53 with p53-binding protein 1 (Huang et al., 2007). As such, there might be an unidentified methyltransferase responsible for K370me2, and this is stimulated by cisplatin and subjected to SMYD2 regulation. Further investigations are required to identify such a methyltransferase (s).

Cisplatin-induced DNA damage not only induces apoptosis, but also cell cycle arrest (Manohar and Leung, 2018). Interestingly, treatment with AZ505 reversed cell cycle arrest as indicated by inhibition of cisplatin-induced p21 upregulation and cyclin D1 downregulation in the kidney during cisplatin injury. Additionally, AZ505 administration promoted proliferation of kidney tubular cells. These data suggests that increased expression and activation of SMYD2 may render kidney tubular cell cycle arrest while inhibition of SMYD2 permits these restrictions and promotes kidney repair and regeneration. Currently, the detailed mechanism by which SMYD2 leads to deregulated regeneration remains unclear. Since p21 is the target gene of p53 in response to DNA damage (Moonen et al., 2018), it is likely that SMYD2 inhibition elicited renoprotection may be due to suppression of p53 activation. However, we cannot exclude the possibility that SMYD2 inhibition may also activate other signaling pathways and genes that lead to kidney tubular cell proliferation.

It is well-documented that pro-inflammatory nature of cisplatin plays a detrimental role in AKI (Holditch et al., 2019). Cytokines/chemokines released by leukocytes and injured TECs are instrumental in initiating and prolonging the extent of inflammation (Holditch et al., 2019). In this study, we found that infiltrated F4/80-positive macrophages and expression of MCP-1 and ICAM-1, two pro-inflammatory factors, were increased in the kidney following cisplatin treatment, and AZ505 significantly reduced these responses. Meanwhile, we found that AZ505 blocked cisplatin-induced phosphorylation of NF-κB and STAT3, two transcription factors associated with kidney inflammation during AKI. Given that both STAT3 and NF-κB are the substrate of SMYD2 in several tumor cells and kidney cyst epithelial cells (Li et al., 2017), we suggest that methylation of STAT3 and NF-κB by SMYD2 may promote cisplatin-induced AKI.

In conclusion, this study was the first to demonstrate that SMYD2 mediates the pathogenesis of AKI following cisplatin treatment. Pharmacological inhibition SMYD2 with AZ505 protects against cisplatin-induced AKI by regulating several key cellular events, including tubular epithelial cell apoptosis and proliferation, and inflammatory response. Nevertheless, SMYD2 may have different functions in response to different injuries and the substrates modified by methylation may also be different in different disease states and different stages of the same disease. Therefore, other studies are required to identify the role and mechanism of SMYD2 in different animal models of AKI.

## Materials and methods

### Chemicals and antibodies

Antibodies to Kim-1, p21 and H3K36me3 were purchased from Abcam Inc. (Cambridge, United Kingdom). Antibodies to cleaved Caspase 3, Cyclin D1, SMYD2, p-p53, p53, p-STAT3, STAT3, p-NFκBp65, and Tubulin were purchased from Cell Signaling Technology (Danvers, MA). Antibodies to PCNA, F4/80, MCP-1, ICAM-1 and TUNEL were purchased from Servicebio (Wuhan, China). Antibodies to NGAL was purchased from R and D SYSTEMS (Minneapolis,United States). Antibodies to GAPDH were purchased from Arigo Biolaboratories Corp. (Shanghai, China). Antibodies to BAX was purchased from Absin Bioscience Inc. (Shanghai, China). Antibodies to Histone-H3 was purchased from Merck (Darmstadt, Germany). Antibodies to NFκBp65 was purchased from Santa Cruz Biotechnology (Santa Cruz, CA). Antibodies to BCL-2 was purchased from Proteintech Group Inc. (Chicargo, United States). SMYD2 siRNA and scramble siRNA were purchased from GenePharma Co.Ltd. (Shanghai, China). AZ505 was purchased from MedChemExpress (New Jersey, United States). Cisplatin and all other chemicals were purchased from Sigma (St. Louis, MO).

### Establishment of mouse acute kidney injury models and AZ505 administration

The cisplatin-induced AKI model was established in male C57/BL6 mouse that weighed 20–25g (Shanghai Super–B&K Laboratory Animal Corp. Ltd.), as described in our previous study (Tang et al., 2018). Briefly, the animals were injected intraperitoneally with a single dose of cisplatin (20 mg/kg) in saline. To investigate the effect of SMYD2 inhibitor AZ505 on AKI, AZ505 (10 mg/kg) in 50 μl of DMSO was given intraperitoneally immediately after cisplatin injection and then administered daily. The dose of AZ505 was selected according to our previous report (Liu et al., 2021). The sham group was injected with an equal volume of DMSO as a control. Six mice were used in each group. Mice were sacrificed at 48h after cisplatin injection. The kidneys were collected for protein analysis and histologic examination. Blood was taken for the measurement of serum creatinine and BUN. All the animal experiments were performed according to the guidelines of the Institutional Animal Care and Use Committee at Tongji University.

### Cell culture and treatment

Human tubular epithelial cells (HK-2) were cultured in Dulbecco’s modified Eagle’s medium (DMEM) with F12 containing 10% fetal bovine serum (FBS), 1% penicillin and streptomycin in an atmosphere of 5% CO2, and 95% air at 37°C. To examine the role of SMYD2 in cisplatin-induced AKI, the small interfering (si) RNA oligonucleotides targeted specially for SMYD2 were used in this study. HK-2 cells were seeded to 30–40% confluence in antibiotic-free medium and then grown for 24 h, followed by SMYD2 siRNA (100 pmol) transfection with lipofectamine 2000. In parallel, scrambled siRNA (100 pmol) was used as control for off-target changes in HK-2 cells. At 24h after transfection, cells were treated with cisplatin (20 μg/ml) for an additional 24h before being harvested for the experiments. Prepared cell lystes were used for immunoblot analysis. All of the *in vitro* experiments were repeated for at least three times.

### Kidney function assay

Kidney function was evaluated by serum creatinine (Scr) and BUN, which were determined by using automatic biochemistry assay (P800, Modular, United States).

### Histochemical and immunofluorescent staining

Histochemical and immunofluorescent staining were carried out according to the procedure described in our previous studies. Formalin-fixed were kidney embedded in paraffin and prepared in 3-μm-thicksections. To evaluate kidney injury, HE staining was performed according to the protocol provided by the manufacture (Sigma-Aldrich). SMYD2, NGAL, F4/80, MCP-1, and ICAM-1 expression in kidney tissue were assessed by immunohistochemical staining. For immunofluorescent staining, the tissue sections were rehydrated and labeled with antibodies for TUNEL and PCNA, then exposed to FITC green (for TUNEL) or red (for PCNA)-labeled secondary antibodies (Servicebio). Slides were viewed with a Nikon Eclipse 80i microscope equipped with a digital camera (DS-Ri1, Nikon, Shanghai, China).

### Immunoblot analysis

Immunoblot analysis of kidney tissue samples and HK-2 cells were conducted as described previously (Tang et al., 2018). The densitometry analysis of immunoblot results was conducted using ImageJ software developed at the national institute of health. The quantification data is given as ratio between target protein and loading control.

### Statistical analysis

All the experiments were conducted at least three times. Data depicted in graphs represent the means ± S.E.M. for each group. For all the experiments, the differences between two groups were made using one-way ANOVA followed by the Tukey test. Statistically significant difference between mean values was marked in each graph. p< 0.05 was considered a statistically significant difference between mean values. The statistical analyses were conducted by using IBM SPSS Statistics 20.0.

## Data Availability

The original contributions presented in the study are included in the article/Supplementary Material, further inquiries can be directed to the corresponding authors.
