# Supplementary material for: Pharmacological inhibition of SMYD2 protects against cisplatin-induced acute kidney injury in mice
Source: Front Pharmacol. 2022 Aug 15;13:829630. doi: 10.3389/fphar.2022.829630 (PMC9421052; doi:10.3389/fphar.2022.829630)
Supplement: Supplementary file 1 [file Presentation1.pptx]

## Slide 1
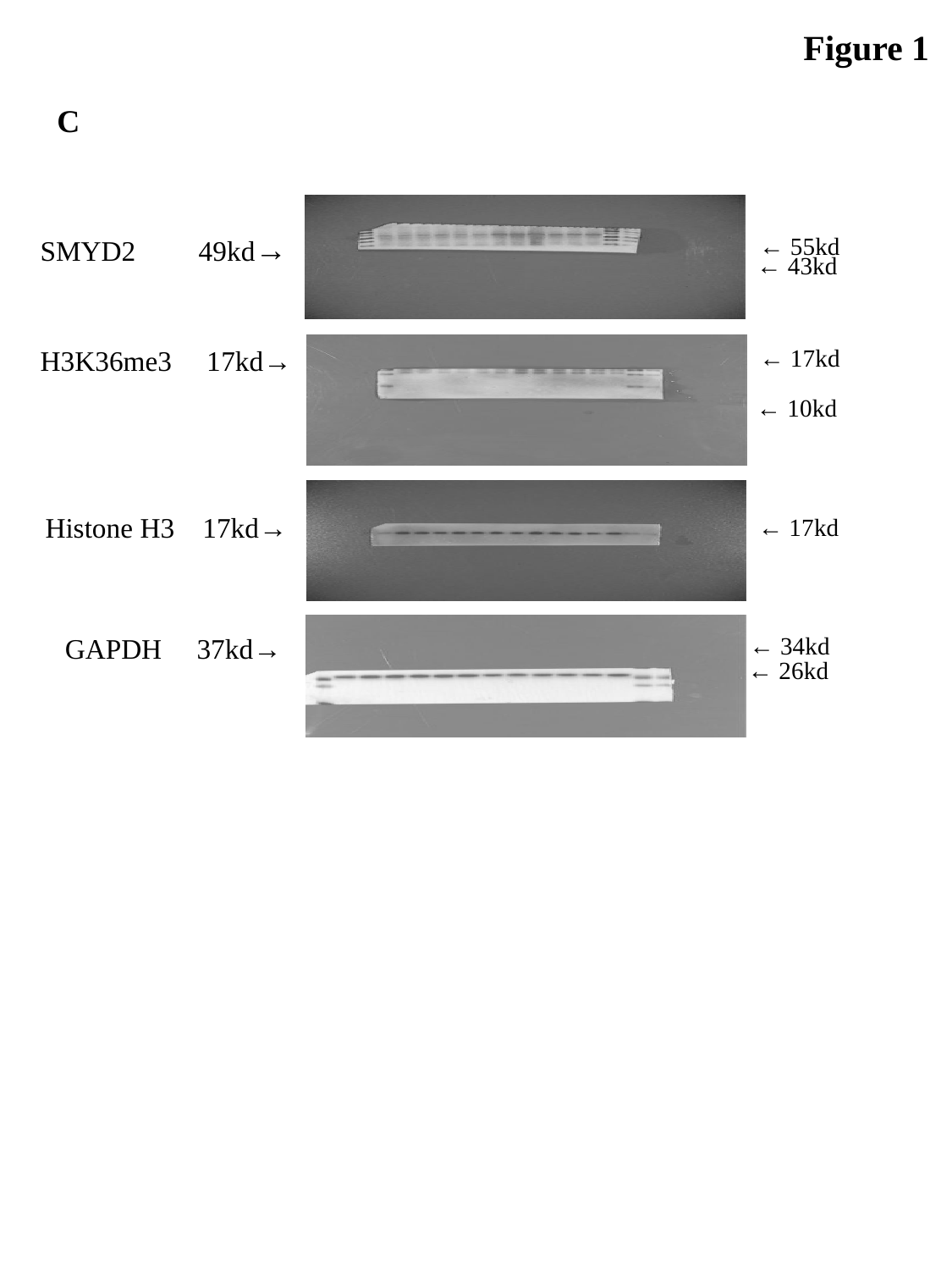

Figure 1
C
 SMYD2 49kd→
← 55kd
← 43kd
 H3K36me3 17kd→
← 17kd
← 10kd
Histone H3 17kd→
← 17kd
← 34kd
GAPDH 37kd→
← 26kd

## Slide 2
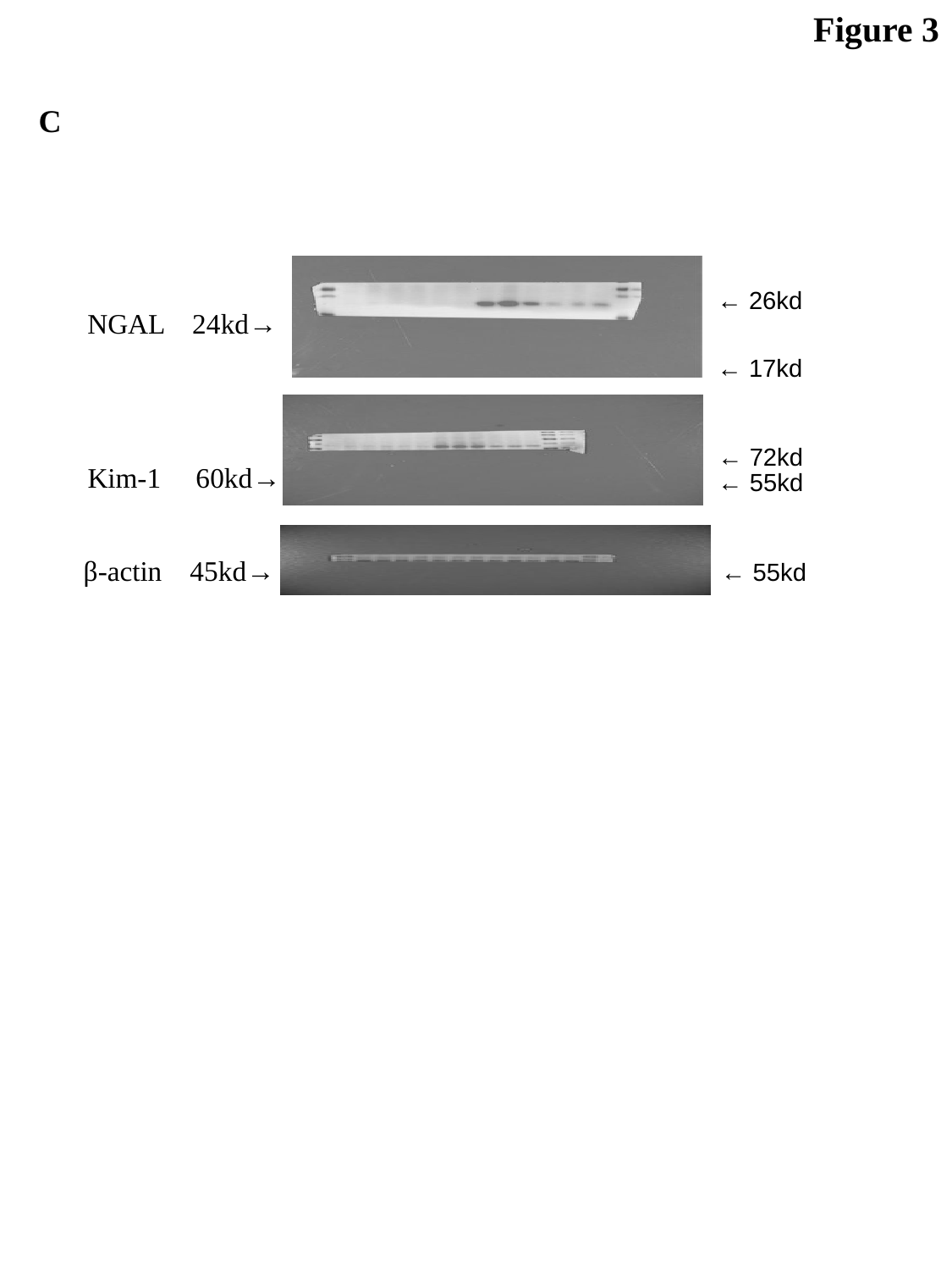

Figure 3
C
← 26kd
 NGAL 24kd→
← 17kd
← 72kd
Kim-1 60kd→
← 55kd
 β-actin 45kd→
← 55kd

## Slide 3
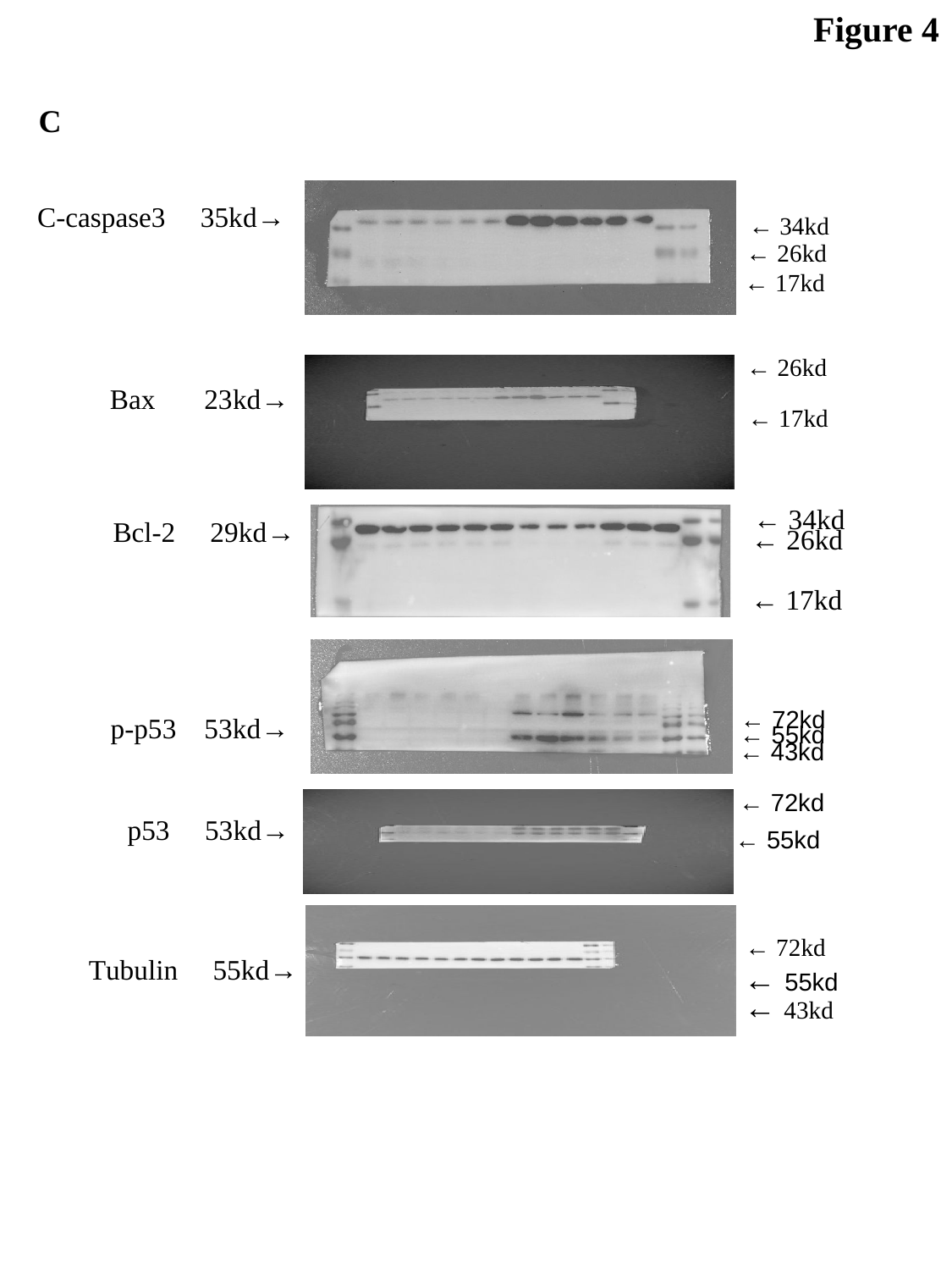

Figure 4
C
C-caspase3 35kd→
← 34kd
← 26kd
← 17kd
← 26kd
 Bax 23kd→
← 17kd
← 34kd
Bcl-2 29kd→
← 26kd
← 17kd
← 72kd
 p-p53 53kd→
← 55kd
← 43kd
← 72kd
 p53 53kd→
← 55kd
← 72kd
Tubulin 55kd→
← 55kd
← 43kd

## Slide 4
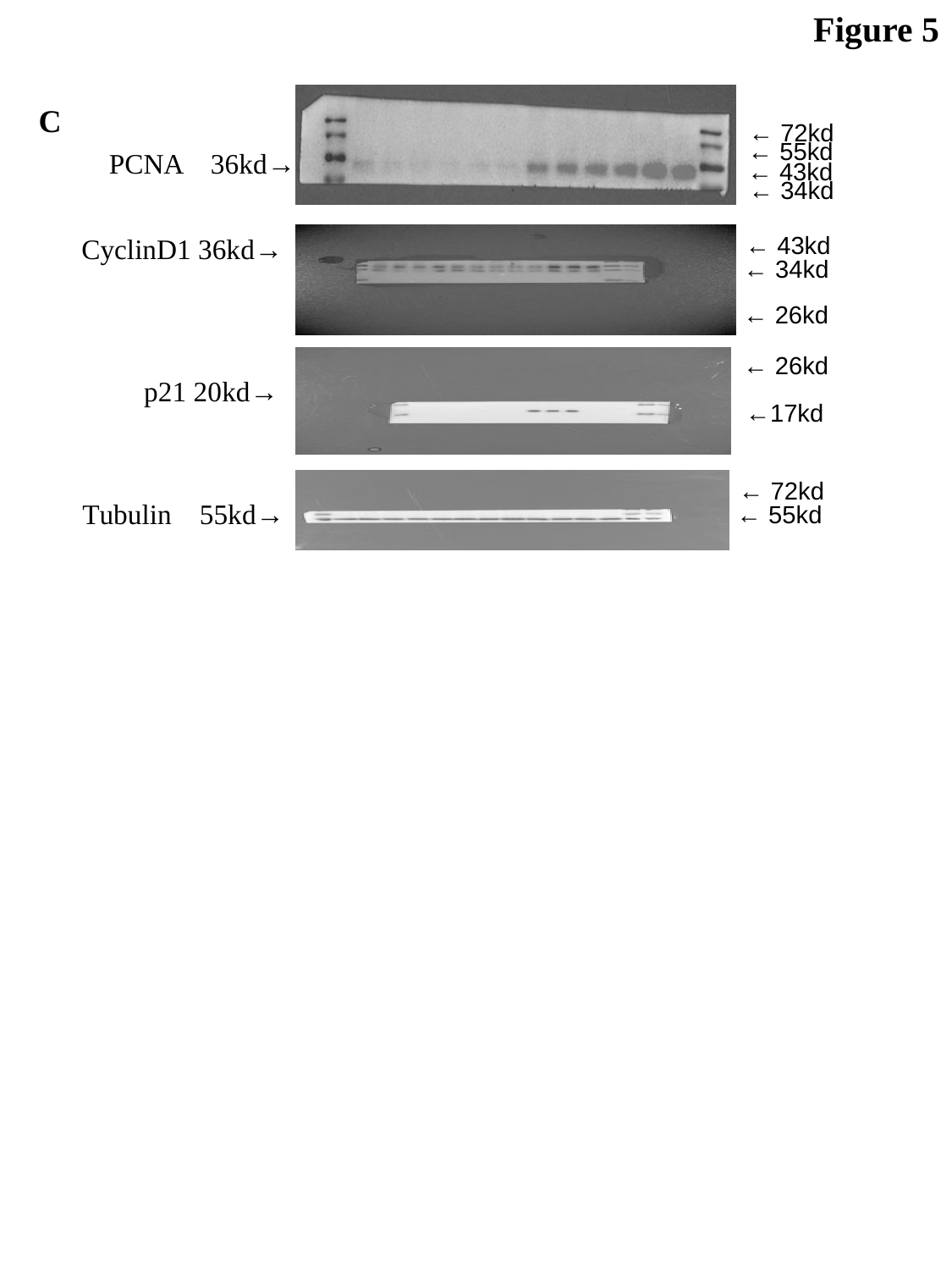

Figure 5
C
← 72kd
← 55kd
 PCNA 36kd→
← 43kd
← 34kd
← 43kd
CyclinD1 36kd→
← 34kd
← 26kd
← 26kd
p21 20kd→
←17kd
← 72kd
Tubulin 55kd→
← 55kd

## Slide 5
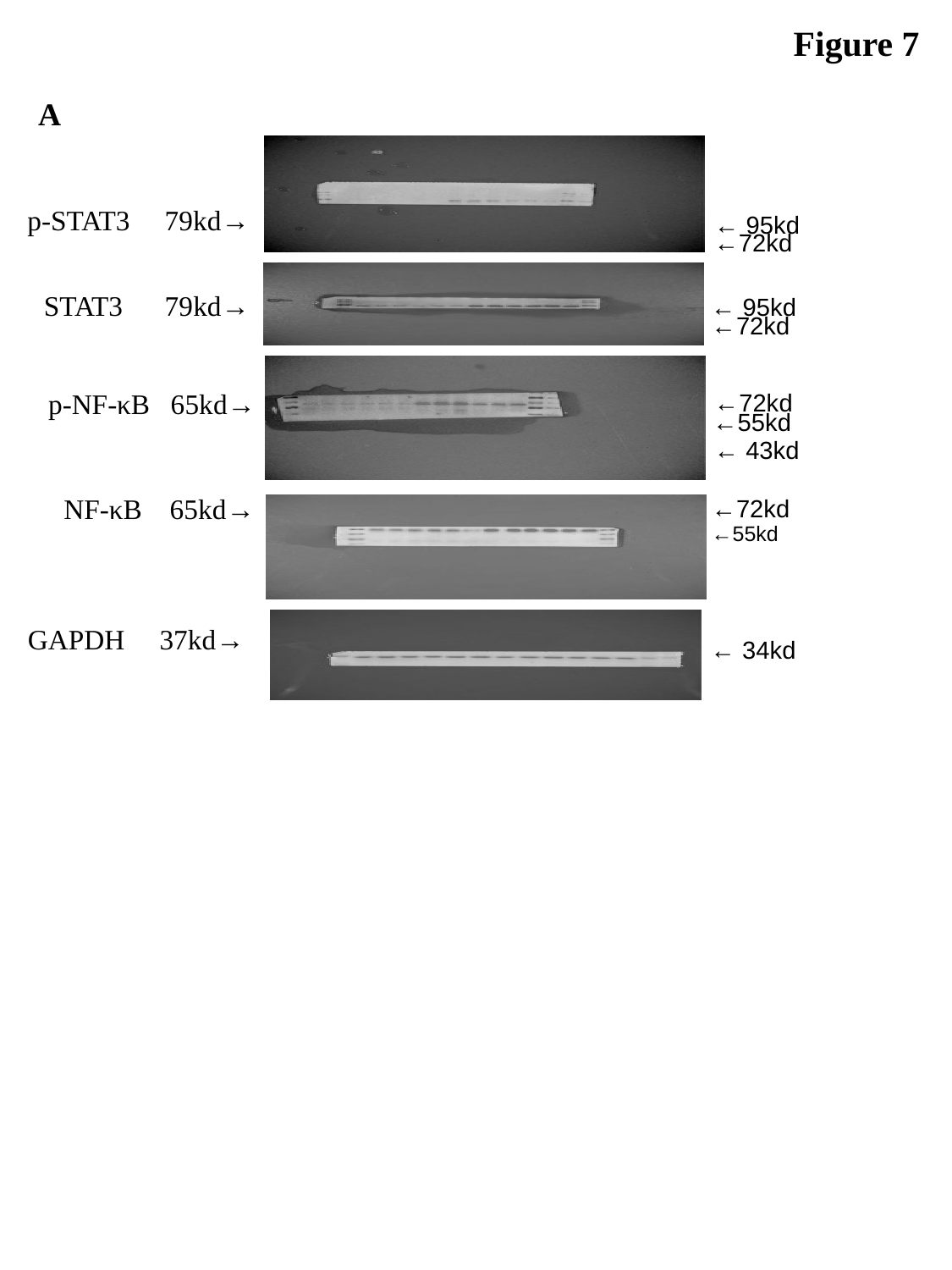

Figure 7
A
p-STAT3 79kd→
← 95kd
←72kd
STAT3 79kd→
← 95kd
←72kd
p-NF-κB 65kd→
←72kd
←55kd
← 43kd
NF-κB 65kd→
←72kd
←55kd
GAPDH 37kd→
← 34kd

## Slide 6
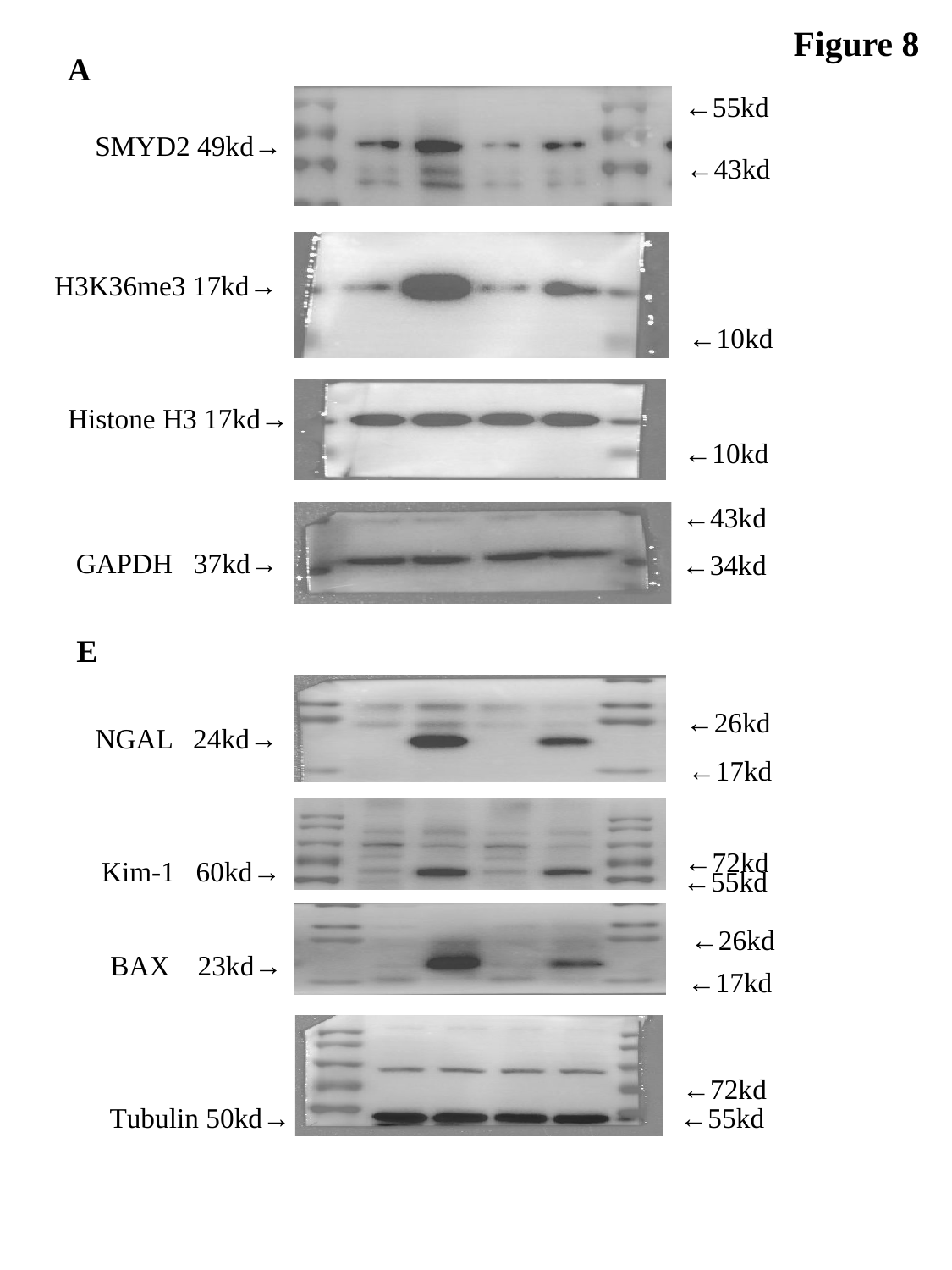

Figure 8
A
←55kd
SMYD2 49kd→
←43kd
H3K36me3 17kd→
←10kd
Histone H3 17kd→
←10kd
←43kd
GAPDH 37kd→
←34kd
E
←26kd
NGAL 24kd→
←17kd
←72kd
Kim-1 60kd→
←55kd
←26kd
 BAX 23kd→
←17kd
←72kd
Tubulin 50kd→
←55kd
